# Streptococcal Toxic Shock Syndrome: A Case Report

**DOI:** 10.7759/cureus.32539

**Published:** 2022-12-14

**Authors:** José Miguel Silva, Joana Gomes Cochicho, Eduardo Carvalho, Ana Rita Parente, Armando Cruz Nodarse, Fernando Pádua

**Affiliations:** 1 Internal Medicine, Hospital Doutor José Maria Grande, Portalegre, PRT; 2 Intensive Care Unit, Hospital Doutor José Maria Grande, Portalegre, PRT

**Keywords:** organ failure from sepsis, immunoglobulins, adsorption cartridges, sorbent cartridge, hemoperfusion, cellulitis, multiorgan system failure, diabetes mellitus type 2, streptococus pyogenes, streptococcal toxic shock syndrome

## Abstract

Streptococcal toxic shock syndrome is a serious complication of group A *Streptococcus* infection with a high mortality rate. Rapid detection, early intensive care support, and surgical management are paramount in treating these patients.

We present a case of a 65-year-old male, with a documented medical history of hypertension, type 2 diabetes mellitus, and peripheral arterial disease. The patient was evaluated in the emergency department with a chief complaint of pain, swelling in his left leg, and fever. Physical examination showed tachycardia, hypotension, and clear inflammatory signs in the left leg. After initial clinical and laboratory evaluation, the patient was admitted with a diagnosis of cellulitis and urinary tract infection. He presented progressive worsening with multi-organ dysfunction, requiring vasopressor support, invasive mechanical ventilation, and renal replacement therapy.

*Streptococcus pyogenes* was isolated in blood cultures, and a streptococcal toxic shock syndrome was considered. Appropriate antibiotic therapy, immunoglobulins, hemoperfusion, and corticosteroid therapy were administered, with clinical improvement. During hospitalization, there was a progressive improvement in the skin lesion. Once clinically stabilized the patient was discharged with follow-up.

The case presented shows the rapid evolution of cutaneous streptococcal infection with multiorgan dysfunction. Although these types of infections have an associated high mortality rate, this patient survived. The use of immunoglobulin and hemoperfusion technique, in this case, might have contributed to this positive outcome. Therefore, we highlight the need for high suspicion of this syndrome, especially in diabetic patients presenting with skin lesions. Once the diagnosis is established, these infections require close surveillance and rapid and intensive treatment.

## Introduction

Streptococcal toxic shock syndrome (STSS) is a serious complication of a streptococcus infection that has a sudden onset, and usually rapidly progresses to multi-organ dysfunction [1]. It has a high mortality rate, even in healthy individuals [2], and is frequently associated with infection by Lancefield group A *Streptococcus* [1]. Clinical manifestations include high fever, hypotension, diffuse erythematous rash, and multiple organ dysfunction, which may rapidly progress to severe and intractable shock. The mechanism of this syndrome is not entirely understood, still, it is believed that a combination of the host response to streptococcal infection and enterotoxins, with superantigen activity, plays a key role [3,4].

The severity of this syndrome and the rapid deterioration of these patients frequently require admission into the Intensive Care Unit (ICU). The treatment usually involves prompt antibiotic administration, infectious source control, and organ failure support [5]. The use of immunoglobulins in the treatment of STSS has been documented, with possible benefits in patient outcomes [5-7]. Hemoperfusion in the context of STSS however is not as well documented, but its use in septic shock is more understood [8].

## Case presentation

We present a case of a 65-year-old male with a documented medical history of hypertension, type 2 diabetes mellitus, and peripheral arterial disease. The patient was seen in the emergency department with a chief complaint of fever (38.7ºC), pain, and swelling in his left leg, which had been worsening in the last three days. Physical examination showed tachycardia (108 beats per minute), hypotension (85/52 mmHg), and clear inflammatory signs in the left leg.

Laboratory tests demonstrated an elevation of C-reactive protein (582 mg/L), serum creatinine (3 mg/dL), and a summary analysis of urine with leukocyturia. Arterial blood gas analysis revealed compensated metabolic acidosis. A non-contrasted computed tomography of the abdomen, pelvis, and left leg showed moderate edema adjacent to the left femoral and iliac vessels.

**Figure 1:**
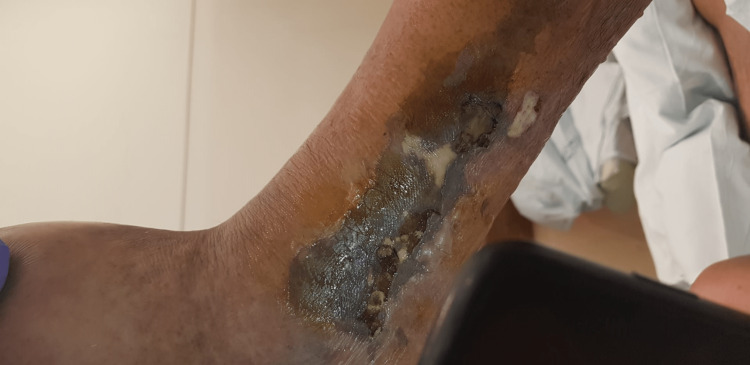
Lesion on the left lower limb on the fifth day of ICU admission

The patient was initially hospitalized for a urinary tract infection and cellulitis of the left lower limb and was medicated with gentamicin and ceftriaxone. However, he remained febrile under antipyretic therapy, and progressed with rapid worsening, evolving into hypercapnic respiratory failure and an altered state of consciousness. The patient evolved with respiratory, hemodynamic, renal, and neurological dysfunction, which required invasive mechanical ventilation, renal replacement technique, and vasopressor support. He was admitted to the ICU on the third day of hospitalization, with a SOFA (sequential organ failure assessment) score of 7 points. At admission into the ICU, antibiotic therapy was escalated to ceftriaxone, gentamicin, and meropenem. *Streptococcus pyogenes* was isolated in blood cultures on the fourth day, and a Streptococcal Toxic Shock Syndrome was assumed (Figure 1).

Antibiotic therapy was therefore switched to linezolid, clindamycin, and penicillin G. Surgical debridement of the skin lesion, in an attempt to control the infection, was performed on the fifth day of hospital admission, after the patient was assumed to be stable enough to undergo this procedure.

Alongside the antibiotic therapy, a three-day course of immunoglobulin therapy was started given evidence, in previously reported cases, of reduced mortality with its use [6,7]. Furthermore, a five-day course of corticosteroid therapy (hydrocortisone 50 mg every six hours) was administered, as the patient was in refractory shock. Both immunoglobulin and corticoid were initiated on the fourth day of hospitalization. 

Even after immunoglobulin and corticoid administration, the patient maintained a high inflammatory state and persistent high fever (40ºC). Given this inflammatory status, it was decided to begin a hemoperfusion technique, in an attempt to reduce inflammatory cytokines. This was started 48 hours after the cessation of the immunoglobulin therapy. For this, a sorbent cartridge model HA380 (Jafron Biomedical, Zhuhai City, China) was used for 72 hours. Venovenous hemodiafiltration was maintained until the normalization of volume and renal function. The patient showed progressive clinical improvement under this treatment.

**Figure 2:**
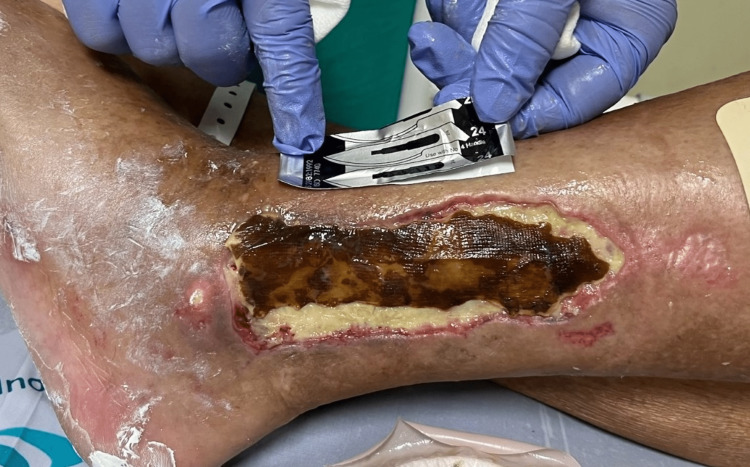
Lesion on the left lower limb, on the 41st day of hospitalization

After a prolonged hospital stay, that included multiple surgical debridements of the skin lesion (Figure 2), and a rehabilitation period in level 1 care, the patient was discharged. Surveillance and nursing care were maintained after discharge.

## Discussion

According to the Global Burden of Disease Project, the prevalence of cellulitis reached 43 million cases in 2019. Antibiotic treatment of the infection is usually effective, with *Streptococcus pyogenes* being one of the most frequent etiological agents [9]. This case highlights the need to request cultural examinations in the case of cellulitis.

There are more than 200 serotypes of *Streptococcus pyogenes* defined from variations in M protein-coding sequences. Some specific serotypes have been shown to have a greater pro-inflammatory capacity, through the formation of complexes between platelets and leukocytes [10]. In the future, the study of serotypes of this agent in clinical practice may help to distinguish infections with the greatest potential for complications, allowing greater surveillance of these patients.

The invasive streptococcal disease presents an incidence of 3.5 cases per 100 000 persons, and an elevated mortality rate [11]. It is more frequent in diabetic patients and advanced ages [12]. The clinical case presented highlights the importance of close surveillance of skin infections in these patients, and their prevention. This group of patients may benefit from the development of a possible future effective anti-streptococcal vaccine [11].

The pathogenic mechanisms of STSS are not fully understood. The host response to streptococcal infection and circulating enterotoxins may contribute to the lethality of this entity [3,4]. Treatment of severe cases, in patients admitted to ICU with multiple organ failure, might benefit from a combination of immunoglobulin and hemoperfusion treatment [4,13]. Although not as widely documented, the use of adsorption cartridges in STSS, might bring a new therapeutic strategy in severe cases. Their use in controlling proinflammatory mediators, that contribute to cellular toxicity and organ dysfunction, might be key in reducing the mortality rate [8].

## Conclusions

STSS is a highly severe clinical entity with a high mortality rate, believed to be in part due to superantigen activity by group A *Streptococcus*. The rapid deterioration of these patients into multiple-organ failure, requiring intensive care and organ support, contributes to the lethality of this syndrome. Future therapeutic strategies involving the use of immunoglobulins and hemoperfusion, alongside prompt antibiotic administration and surgical debridement, might help reduce the high mortality rate.

The case presented exemplifies the level of care and the need for organ support in these patients. Moreover, it demonstrates the use of adsorption cartridges in the case of STSS, with a good clinical outcome. We emphasize the need to prevent this type of infection, and the rapid suspicion of this syndrome, especially in diabetic patients.
